# The problems of meta-analysis for antibiotic treatment of chronic obstructive pulmonary disease, a heterogeneous disease: a commentary on Puhan et al

**DOI:** 10.1186/1741-7015-6-29

**Published:** 2008-10-10

**Authors:** Sanjay Sethi

**Affiliations:** 1 Division of Pulmonary, Critical Care and Sleep Medicine, University at Buffalo, State University of New York and VA Western New York Healthcare System, Buffalo, New York, USA

## Abstract

Exacerbations are a major cause of morbidity and mortality in chronic obstructive pulmonary disease. Exacerbations can be of bacterial, viral or mixed etiology, with bacteria involved in 50% of exacerbations. Consequently, current management of exacerbations frequently involves the use of antibiotics. The paper by Puhan et al published this month in *BMC Medicine *examines the benefit of antibiotics in placebo-controlled trials in mild to moderate outpatient exacerbations. The authors use a meta-analytic approach and rightly conclude that more trials are needed in this area. However, the heterogeneity of chronic obstructive pulmonary disease patients and exacerbations and the limited end-points in past trials do not allow firm conclusions to be drawn about antibiotic use in outpatient exacerbations based on this meta-analysis. Future trials need to take into account this heterogeneity as well as incorporate novel end-points to address this important issue.

## Background

Bronchitis is among the most common reasons for antibiotic prescription worldwide. This diagnosis includes two distinct entities, acute bronchitis in the absence of underlying lung disease and acute exacerbations of chronic bronchitis or chronic obstructive pulmonary disease (COPD). Acute bronchitis is predominantly a viral disease with good evidence that antibiotics are not of benefit in its management. Exacerbations of COPD, on the other hand, can be of bacterial, viral or mixed etiology, with bacterial infection currently estimated to contribute up to 50% of exacerbations. Furthermore, in contrast to acute bronchitis, these exacerbation episodes are not benign events, as they have consequences ranging from days lost from work, deterioration of health status, progression of airflow obstruction and even death. Therefore, appropriate management of COPD exacerbations is crucial.

Antibiotics are often used in the management of COPD exacerbations. It is estimated that more than 80% of COPD exacerbations are treated on an outpatient basis, which can be regarded as mild to moderate exacerbations. The article published by Puhan et al this month in *BMC Medicine *makes the argument that there is a lack of evidence for antibiotic benefit in mild to moderate exacerbations of COPD and that additional placebo-controlled trials are required [1]. Their argument is based on a meta-analytic approach where they identified five placebo-controlled randomized trials in the literature, which were confined to 'mild to moderate' exacerbations of COPD. With this approach, although all but one of the trials favor antibiotics, the combined odds ratio is not statistically significant.

Although their conclusions based on the analysis are not erroneous, their approach to this question highlights a number of issues that make it difficult to resolve this argument; these include the heterogeneity of exacerbations, patients and antibiotics in these trials. Another major issue is the acceptance of the results of these trials without critically examining the limitations of the end-points used in these studies. The 'devil is in the details' in these trials, and these details are often overlooked in a meta-analytic approach.

## Severity of exacerbations

Puhan et al have grouped together mild and moderate exacerbations based on the site of treatment, that is, outpatient treatment. This classification is clearly very broad as the site of care will vary among countries and healthcare systems as well as with patient and physician preferences. Furthermore, over time, changes in healthcare delivery and results of outcome studies can change the site of care for the same severity of exacerbation. A 40-year-old smoker without underlying airway obstruction, infrequent exacerbations and free of comorbid conditions would have been included as a 'mild to moderate' exacerbation. On the other hand, a patient with severe COPD, frequent exacerbations and comorbid conditions who does not require hospitalization would also be classified as a 'mild to moderate exacerbation'. In the former patient, it is possible that host immunity can adequately deal with the infection and the exacerbation will spontaneously resolve. In the latter patient, such resolution is less likely and complications are more frequent. Grouping these patients together can lead to confusing and contradictory results.

The severity of an exacerbation is a complicated concept, constituted by at least two factors, the severity of the underlying COPD and the acute change induced by the exacerbation itself. Therefore, a patient with severe underlying COPD will have significant clinical consequences from a relatively small change from the baseline state, while a patient with mild COPD will tolerate a much larger change in symptoms and lung function. It is evident that we need more objective measures of severity of exacerbations. Ongoing developmental efforts in patient-reported outcomes and biomarkers should provide us with such tools in the future, and allow for trials to be pooled as attempted by Puhan et al here.

## Heterogeneity of COPD

COPD is a heterogeneous disease. Outcomes of exacerbations worsen and antibiotic benefit in exacerbations increases with worsening underlying airflow obstruction, in frequent 'exacerbators' and with comorbid conditions [2,3]. This is likely related to a greater proportion of bacterial etiology and more severe local immunocompromise in these patients.

In grouping together the trials in their analysis, Puhan et al have pooled together patients who are very heterogeneous with respect to their COPD disease and, therefore, could not discern a beneficial effect of antibiotics. This is best illustrated by comparing the patient populations of two trials included in their analysis; the trial conducted by Anthonisen et al [4], which showed a significant benefit of antibiotics, and the trial conducted by Sachs et al [5], which failed to show benefit (Table 1). (Note: In the Anthonisen study [4], there was significant benefit with antibiotics when all 362 exacerbations were considered. For their analysis, Puhan et al chose to consider only the first exacerbation from the 116 patients in the study, with considerable alteration in the results). As is evident from this comparison, Sachs et al [5] included patients of younger age, mild underlying disease and asthma. Not surprisingly, only 11% of their exacerbations were associated with a positive bacterial culture, rather than the usual 40% to 50%. Not surprisingly, antibiotics were of no benefit in this study and, in their placebo arm, there was a 93% resolution rate compared with 55% in the Anthonisen study [4].

**Table 1:** Comparison of patients included in two placebo-controlled trials of antibiotics in acute exacerbation of chronic bronchitis

**Characteristic**	**Anthonisen**	**Sachs**
*N*	362	71
Age in years (mean ± standard deviation)	67.3 ± 9	51.7 ± 16.3
Minimum age for inclusion in years	35	18
Smoking in pack-years (mean ± standard deviation)	39.9 ± 28.9	16.5 (0.15–77)
Smokers (% of subjects)	93.6	69.1
Asthmatics	Excluded	Included
Forced expiratory volume_1 _(% predicted)	33.9 ± 3.7	NA
Peak expiratory flow (liter/minute)	227.5 ± 96.1	285.3 ± 99.2

Data from Anthonisen et al [4] and Sachs et al [5].

## All antibiotics are not the same

An additional consideration is the spectrum of the different antibiotics used to treat COPD. In this study, the authors tended to treat all antibiotics as equivalent when used to treat exacerbations of COPD. Antibiotics do differ in their antimicrobial spectrum, pharmacokinetic/pharmacodynamic profiles and ability to penetrate respiratory tissues. Recent studies indeed show differences in clinical outcomes among antibiotics used in exacerbations. A recent meta-analysis of antibiotic comparison trials, which were quite homogenous, demonstrated that amoxicillin results in suboptimal outcomes with increased risk of clinical failures in COPD [6]. This has been seen particularly since the early 1990s, when resistance emerged to this agent. Interestingly, two trials (Sachs et al [5] and Jorgensen et al [7]) included in the analysis by Puhan et al, both not showing a significant benefit of antibiotics, used amoxicillin and were conducted in the 1990s. Two trials comparing fluoroquinolones with non-fluoroquinolone antibiotics, the GLOBE and MOSAIC trials, showed more complete clinical resolution of exacerbations and a prolonged time to the next exacerbation [8,9].

## End-points in exacerbation trials

Analysis of any study should critically examine if its end-points were adequate to demonstrate the potential benefits of the intervention being tested and were clinically relevant. Unfortunately, in the studies evaluated by Puhan et al, as well as in the vast majority of antibiotic comparison trials in exacerbations of COPD, end-points used favor the demonstration of equivalence rather than differences among the arms (Table 2) [10]. Partly, this is the result of mandates (now obsolete) by regulatory agencies, such as the Food and Drug Administration (FDA). These end-points assessed at 2 to 3 weeks after the onset of symptoms and the initiation of therapy miss differences in therapeutic effect earlier during the course of treatment. In addition, these end-points have minimal relevance to clinical practice. In clinical practice, most physicians and patients expect clinical improvement in their exacerbation at 3 to 5 days after initiation of treatment. In fact, with insufficient improvement in that timeframe, therapy is often altered or expanded. Allegra et al [11] did conduct a placebo-controlled trial where they used a 5-day time-point, showing a substantial benefit of antibiotics, which was excluded from the analysis by Puhan et al.

**Table 2:** Limitations of published placebo-controlled antibiotic trials in acute exacerbations of chronic obstructive pulmonary disease

**Limitation of study design**	**Potential consequences**
Small number of subjects	Type 2 error
Subjects with mild or no underlying chronic obstructive pulmonary disease included	Diminished overall perceived efficacy of antibiotics
Non-bacterial exacerbations included	Type 2 error
End-points compared at 3 weeks after onset	Spontaneous resolution mitigates differences between arms
	Clinically irrelevant as most decisions about antibiotic efficacy are made earlier
Speed of resolution not measured	Clinically relevant end-point not assessed
Lack of long-term follow-up	Time to next exacerbation not assessed
Antibiotic resistance to agents with limited *in vitro *antimicrobial efficacy	Diminished overall perceived efficacy of antibiotics
Poor penetration of antibiotics into respiratory tissues	Diminished overall perceived efficacy of antibiotics
Concurrent therapy not controlled	Undetected bias in use of concurrent therapy

Reproduced with permission from Sethi [10].

The adequacy of the traditional goals of treatment of an exacerbation, recovery to baseline clinical status and the prevention of complications, are being questioned because of several new observations. These include realization of the importance of exacerbations in the course of COPD, the role of infection in exacerbations, the high rates of relapse with an adequate initial clinical response, and the role played by chronic infection in the pathogenesis of COPD. Today, confining our goal in the treatment of COPD exacerbations to short-term resolution of symptoms would be analogous to treating acute myocardial infarction with the only aim being resolution of chest pain.

Table 3 lists several other important goals of treatment, both clinical and biological, that should be considered [12]. In fact, the FDA wants precise symptom measurement with a patient-reported outcome measure as the major end-point of future studies of antibiotics in exacerbations. Practical application of the biological goals of treatment of exacerbations should be feasible in the future with ongoing development of simple, rapid and reliable measurements of inflammation and infection.

**Table 3:** Proposed goals of treatment of chronic obstructive pulmonary disease exacerbation

**Goals**	**Comments**
**Clinical**	
Clinical resolution to baseline	Needs baseline assessment prior to exacerbation onset for comparison
Prevention of relapse	Relapse within 30 days is quite frequent
Increasing exacerbation-free interval	Needs long-term follow-up after treatment
Faster resolution of symptoms	Needs validated symptom assessment tools
Preservation of health-related quality of life	Sustained decrements seen after exacerbations
	
**Biological**	
Bacterial eradication	Often presumed in usual antibiotic comparison studies
Resolution of airway inflammation	Shown to be incomplete if bacteria persist
Resolution of systemic inflammation	Persistence of systemic inflammation predicts early relapse
Restoration of lung function to baseline	Incomplete recovery is seen in significant proportion
Preservation of lung function	Needs long-term studies

## Conclusion

Recent American College of Physicians/American College of Chest Physicians guidelines for COPD exacerbations state for future research priorities: 'Our first research objectives must include untangling the questions surrounding selection of patients for antibiotic and corticosteroid treatment, identifying optimal dosing and durations for these agents, and determining the degree to which broad- and narrow-spectrum antibiotics have similar efficacy' [12]. Undoubtedly, as stated by Puhan et al, we need to enlarge our evidence base for the treatment of exacerbations with placebo-controlled trials. However, as highlighted in this commentary, these trials should use contemporary end-points so that we do not miss important, clinically relevant benefits of antibiotics, not assessed by traditional end-points.

Until such studies are completed, how should we treat outpatient 'mild to moderate' exacerbations? Lack of evidence is not the same as lack of efficacy. As discussed above, the heterogeneity of severity and patients among outpatient exacerbations demonstrates that grouping them all together is perhaps not the best course of action. 'Moderate' exacerbations, such as those included in the Anthonisen [4] trial, should receive antibiotics. The choice of initial antibiotics in these patients should be based on a 'risk stratification' approach. 'Mild' exacerbations, such as those included in the Sachs [5] trial, likely do not need antibiotics, especially if they do not have purulent sputum. These are the patients that should be included in placebo-controlled trials with contemporary end-points.

## Abbreviations

COPD: chronic obstructive pulmonary disease; FDA: Food and Drug Administration.

## Pre-publication history

The pre-publication history for this paper can be accessed here:



## References

[B1] Puhan MA, Vollenweider D, Steurer J, Bossuyt P, ter Riet G (2008). Where is the supporting evidence for treating mild to moderate chronic obstructive pulmonary disease patients exacerbations with antibiotics?: a systematic review. BMC Medicine.

[B2] Miravitlles M, Murio C, Guerrero T (2001). Factors associated with relapse after ambulatory treatment of acute exacerbations of chronic bronchitis. DAFNE Study Group. Eur Respir J.

[B3] Wilson R, Jones P, Schaberg T, Arvis P, Duprat-Lomon I, Sagnier PP (2006). Antibiotic treatment and factors influencing short and long term outcomes of acute exacerbations of chronic bronchitis. Thorax.

[B4] Anthonisen NR, Manfreda J, Warren CPW, Hershfield ES, Harding GKM, Nelson NA (1987). Antibiotic therapy in exacerbations of chronic obstructive pulmonary disease. Ann Intern Med.

[B5] Sachs APE, Koeter GH, Groenier KH, Waaij D van der, Schiphuis J, Jong BMd (1995). Changes in symptoms, peak expiratory flow, and sputum flora during treatment with antibiotics of exacerbations in patients with chronic obstructive pulmonary disease in general practice. Thorax.

[B6] Dimopoulos G, Siempos II, Korbila IP, Manta KG, Falagas ME (2007). Comparison of first-line with second-line antibiotics for acute exacerbations of chronic bronchitis: a metaanalysis of randomized controlled trials. Chest.

[B7] Jorgensen AF, Coolidge J, Pedersen PA, Petersen KP, Waldorff S, Widding E (1992). Amoxicillin in treatment of acute uncomplicated exacerbations of chronic bronchitis. A double-blind, placebo-controlled multicentre study in general practice. Scand J Prim Health Care.

[B8] Wilson R, Schentag JJ, Ball P, Mandell L (2002). A comparison of gemifloxacin and clarithromycin in acute exacerbations of chronic bronchitis and long-term clinical outcomes. Clin Ther.

[B9] Wilson R, Allegra L, Huchon G, Izquierdo JL, Jones P, Schaberg T, Sagnier PP (2004). Short-term and long-term outcomes of moxifloxacin compared to standard antibiotic treatment in acute exacerbations of chronic bronchitis. Chest.

[B10] Sethi S (2004). Bacteria in exacerbations of chronic obstructive pulmonary disease. Phenomenon or epiphenomenon?. Proc Am Thorac Soc.

[B11] Allegra L, Blasi F, de Bernardi B, Cosentini R, Tarsia P (2001). Antibiotic treatment and baseline severity of disease in acute exacerbations of chronic bronchitis: a re-evaluation of previously published data of a placebo-controlled randomized study. Pulm Pharmacol Ther.

[B12] Bach PB, Brown C, Gelfand SE, McCrory DC (2001). Management of acute exacerbations of chronic obstructive pulmonary disease: a summary and appraisal of published evidence. Ann Intern Med.

